# Deterioration Behavior of Concrete Beam Reinforced with Carbon Fiber-Reinforced Plastic Rebar Exposed to Carbonation and Chloride Conditions

**DOI:** 10.3390/polym17010055

**Published:** 2024-12-29

**Authors:** Seung-Yun Lee, Sun-Hee Kim, Wonchang Choi

**Affiliations:** Department of Architectural Engineering, Gachon University, Seongnam-si 13120, Republic of Korea; dltmddbs1231@gachon.ac.kr (S.-Y.L.); shkim6145@gachon.ac.kr (S.-H.K.)

**Keywords:** exposure experiment, carbon fiber-reinforced polymer, chloride environment, durability test, carbonation test

## Abstract

The absence of carbon fiber-reinforced rebar performance standards in Korea has limited its reliability. This study investigates the durability performance of carbon fiber-reinforced polymer rebar as an alternative to traditional steel reinforcement in concrete structures. Concrete beams reinforced with carbon fiber-reinforced polymer rebar were exposed to chloride environments for durations of 35 and 70 days and then subjected to bending tests to evaluate their durability. The results demonstrate that the strong bond between the carbon fiber-reinforced polymer and concrete effectively prevented brittle fracture, even under exposure to harsh chloride. A scanning electron microscope analysis of the specimens exposed to chloride showed no deterioration of the carbon fiber-reinforced polymer rebar, highlighting its exceptional resistance to corrosion. Furthermore, durability tests were conducted in a carbonation chamber for 8 and 12 weeks, with no signs of degradation in the carbon fiber-reinforced polymer rebar. These findings suggest that carbon fiber-reinforced polymer rebar offers excellent resistance to both chloride-induced corrosion and carbonation, making it a promising solution to enhance the longevity and durability of reinforced concrete structures exposed to aggressive environmental conditions.

## 1. Introduction

Steel rebar corrosion occurs due to factors like exposure to chlorine, alkaline or acidic conditions, and moisture. It exacerbates the cracking of concrete and accelerates aging, significantly affecting the durability of reinforced concrete structures. To address these issues, recent research in South Korea has focused on utilizing fiber-reinforced plastic (FRP) rebars and grids as alternatives to traditional reinforcement materials.

FRP exhibits exceptional impermeability and corrosion resistance, allowing for semi-permanent usage. It also demonstrates superior strength and alkali resistance compared to reinforced concrete [1,2]. Furthermore, FRP offers advantages such as extending the lifespan of structures, enhancing durability, reducing maintenance costs, and decreasing self-weight [3].

Ahmed et al. [4] demonstrated that concretes reinforced with glass FRP (GFRP) and basalt FRP (BFRP), using seawater and marine sand, were suitable for marine environments in terms of durability and economic viability. Wu et al. [5] showed that carbon FRP (CFRP) and GFRP maintained adequate strength under prolonged corrosion in marine environments; however, BFRP exhibited accelerated degradation in highly alkaline conditions, with its residual strength falling below the standard. Wang et al. confirmed the behavior and deterioration mechanisms of RC beams in a chloride–sulfate environment through experimental investigation, establishing that chloride and sulfate ions affected the behavior of corroded RC beams. Through experimentation, Zhou et al. [6] demonstrated that FRP bars exhibited similar ductility to concrete beams utilizing natural aggregates, albeit with slightly lower flexural strength in SSRC beams. Liu and Fan [7] studied the effects of CFRP reinforcement and chloride corrosion on the flexural behavior of prestressed concrete beams. After reinforcement with CFRP, the beams were immersed in chloride for 120 days. The results of the flexural test show that the cracking load increased, and the stiffness reduction rate decreased. Hawileh et al. [8] studied the bond strength and durability of concrete reinforced with CFRP composites and galvanized steel using epoxy adhesive and cementitious mortar. Concrete specimens were reinforced with CFRP and galvanized steel. Durability was measured after exposure to salt water and direct sunlight for 28 and 540 days. CFRP-reinforced specimens showed the least degradation in load-carrying capacity in both control regimes. Li et al. [9] tested the durability of FRP–concrete joint systems immersed in a chloride solution. The FRPs used were CFRP and BFRP, and they were immersed in a 5% NaCl solution at 40 °C. The immersion time was set to 90, 180, 270, and 360 days to determine the effect on mechanical properties. As the immersion time elapsed, the tensile strength of CFRP increased and then decreased due to the hardening of the epoxy resin. CFRP showed better chloride resistance than BFRP, and the strength retention rate of CFRP after 360 days was approximately 160% higher than that of BFRP. Li et al. [10] experimentally studied the durability of CFRP grids in a seawater environment. After exposure to water and seawater for 360 days, the tensile strength of the CFRP grid tended to decrease by 6.2% in water and 10.2% in seawater. Lu et al. [11] performed tensile strength tests on CFRP, GFRP, and BFRP bars after exposure to alkaline, seawater, and water for 45, 90, 135, and 180 days. CFRP showed the greatest performance degradation in seawater, with a tensile strength decrease of up to 21% after 180 days. Benmokrane et al. [12] conducted tensile strength and shear strength tests to evaluate the durability of fiber composites in alkaline environments. The experimental results show that the tensile strength and shear strength of carbon fibers exposed to alkaline environments were similar to those of unexposed carbon fiber specimens, indicating that the carbon fibers were acceptable for exposure to alkaline environments. Rosa et al. [13] investigated the bonding properties between concrete and GFRP rebar at high temperatures, demonstrating significant decreases in the stiffness and strength of GFRP when subjected to high-temperature pull-out tests up to 300 °C. Ji et al. [14] conducted experiments to assess the durability characteristics of concrete reinforced with CFRP, dividing the study into three segments—basic, partial, and full reinforcement—to evaluate performance variations under combined chlorine salt and salt freeze conditions. At identical exposure times, the chloride ion concentration in the standard specimen was 200 times greater than that in the fully reinforced specimen. After 100 freeze–thaw cycles, the strength reduction rate was 50% for the standard specimen and 10% for the CFRP-reinforced specimen.

Al-Khafaji et al. subjected six CFRP sheet-reinforced concrete beams to saline and tap water immersion under outdoor environmental conditions for durations of 60 and 195 d, subsequently conducting pull-off shear and three-point bending tests. Tap water immersion had a greater impact on adhesion strength and CFRP than saltwater immersion, with an average strength reduction after 60 d of 26% for tap water and 5% for saltwater [15]. Dong et al. conducted experiments on the flexural durability of seawater–sand concrete beams reinforced with rebars, stainless steel, and CFRP bars. The FRP-reinforced beam showed a 30% reduction in load capacity and up to a 2.2-fold increase in crack width due to the reduced bond strength with BFRP stirrups [16]. Lee et al. [17] highlighted that the CFRP rebar required only 50% of the reinforcement typically required for general reinforced concrete columns, excluding the rebar ratios exceeding 4%. However, for rebars below this threshold, the cross-section size and rebar quantity can be reduced when using the CFRP rebar. Choi et al. [18] compared the flexural strength, deflection, and crack width of concrete beams reinforced with rebar and CFRP rebar. The results demonstrate that using the CFRP rebar yielded more pronounced design flexural strength than the other rebar despite using the same amount. Therefore, CFRP rebar is a suitable substitute for rebar. However, the mechanical properties of FRP materials vary depending on the weaving method. Examining FRP’s durability and mechanical properties is crucial for effectively applying it to structures. Most studies on the durability of FRP have focused on concrete beams reinforced with FRP sheets or on concrete beams with main components reinforced with steel [19,20,21]. In contrast, research is lacking on the use of CFRP rebars as a substitute for traditional reinforcement. Therefore, to evaluate the durability of concrete beams reinforced with CFRP rebars developed as a substitute for rebars, this study conducted bending tests on concrete beams exposed to a chloride environment and carbonization.

## 2. Mechanical Properties

### 2.1. Tensile Properties of CFRP

**Figure 1:**
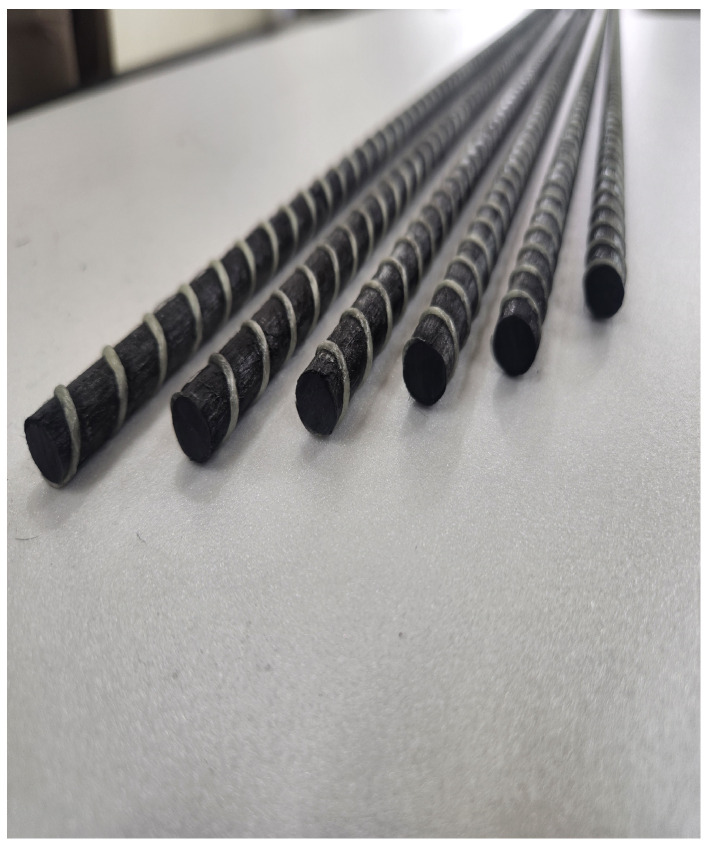
Tensile specimen.

To ascertain the tensile characteristics of CFRP rebar, developed as a substitute for steel reinforcement, the diameter of the CFRP rebar was fabricated to match that of the commonly used D10 steel rebar, and tensile strength tests were conducted in accordance with ASTM D 7205 standards [22]. The developed CFRP rebar is shown in Figure 1.

The tensile specimen had a total length of 1900 mm, with a steel pipe fixed at both ends: one end was 500 mm, and the other was 700 mm, filled with non-shrinkage mortar.

**Figure 2:**
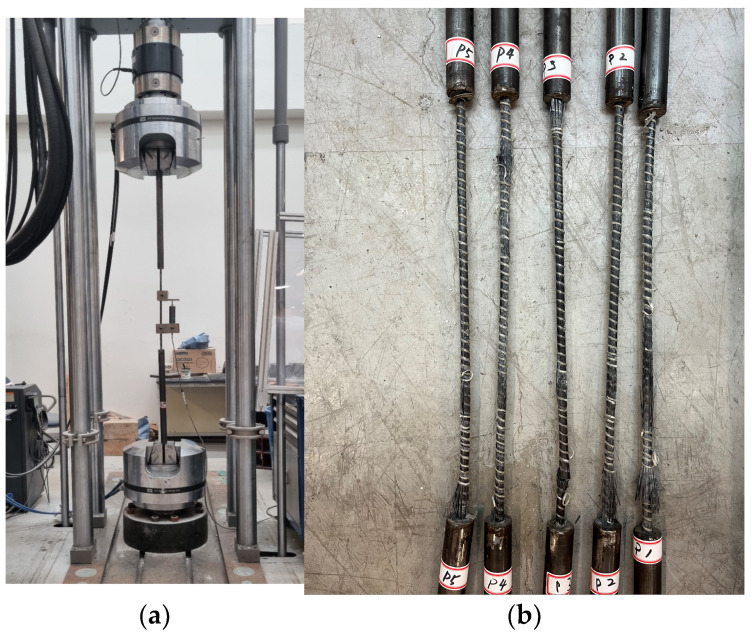
Tensile test set-up and failure mode. (**a**) Test set-up; (**b**) failure mode.

The tensile strength test was performed using a servo-hydraulic tensile strength testing machine with a capacity of 1.2 MN, and the loading rate was set at 5 mm/min. Figure 2a provides an overview of the tensile strength test.

The tensile strength test results indicate that most specimens failed at the central region, as shown in Figure 2b.

The tensile strength and modulus of elasticity in Table 1 were calculated using Equations (1) and (2).

(1) $$\sigma =\frac{P}{A},$$

where P is the maximum load applied to the specimen and A is the cross-sectional area of the specimen.

(2) $$E=\frac{\sigma_{\Delta }}{\varepsilon_{\Delta }},$$

Here, $\varepsilon_{\Delta }$ is the difference between the 0.001 and the 0.006 strains, and $\sigma_{\Delta }$ is the stress difference at each strain.

The tensile strength test results show that the tensile stress and modulus of elasticity of D10 carbon-reinforced steel were 2300 Mpa and 134 Gpa, respectively.

**Figure 3:**
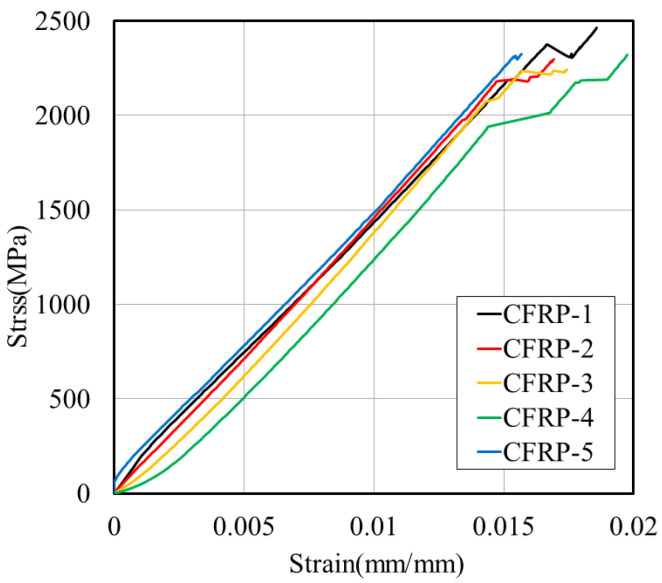
Stress–strain relationship of CFRP tensile strength test.

Figure 3 presents the stress–strain graph from the tensile strength test, and Table 1 provides the results.

### 2.2. Concrete

#### 2.2.1. Concrete Mix Design

The concrete mix design was formulated with high strength to prevent flexural failure of the CFRP reinforcement bars. The water/cement ratio was set at 33%, the maximum aggregate size at 25 mm, and the air content at 2%. The mix proportions of the test specimens are outlined in Table 2.

#### 2.2.2. Compressive Strength Test for Concrete

**Figure 4:**
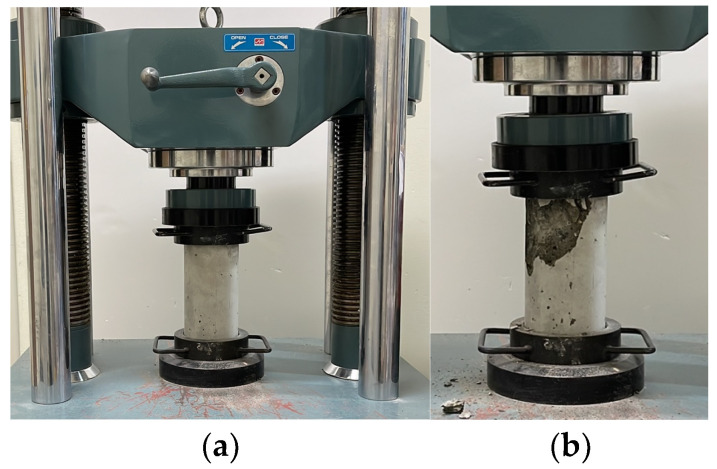
Compressive strength test for concrete. (**a**) Test set-up; (**b**) failure mode.

A compressive strength test was conducted to analyze the mechanical properties of the concrete. The specimen dimensions were 100 mm in diameter and 200 mm in length, and a universal testing machine with a load capacity of 1000 kN was used. The loading rate was set at 1 mm/min. Figure 4a illustrates the compressive strength test, while Figure 4b depicts the failure modes of the concrete. The results of the compressive strength test are presented in Table 3.

## 3. Experiment Plan and Method

### 3.1. Fabrication of Specimens

#### 3.1.1. Chloride Specimens

**Figure 5:**
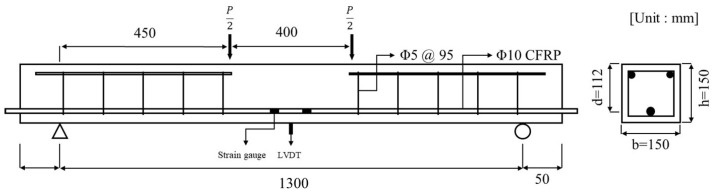
Details of chloride specimen.

The factors that influence the bond strength between concrete and CFRP (such as the type of bar, surface treatment, and concrete cover) also impact the global behavior of the beam. This aspect is critical, especially in service conditions, in terms of crack width and deflection. Specimens were fabricated to examine the effects of CFRP rebar in concrete exposed to a chloride environment. We examined the behavior of reinforced concrete beams to determine the effects of CFRP rebar on their performance under these conditions. Chloride specimens were fabricated as concrete beams measuring 1400 mm in length, 150 mm in width, and 150 mm in height, as illustrated in Figure 5. The compressive strength of the concrete was 56.24 MPa, and D10 CFRP rebar was used for reinforcement. All sections, except the flexural section, were reinforced against shear with D8 rebars spaced at 95 mm intervals.

#### 3.1.2. Carbonation Specimens

**Figure 6:**
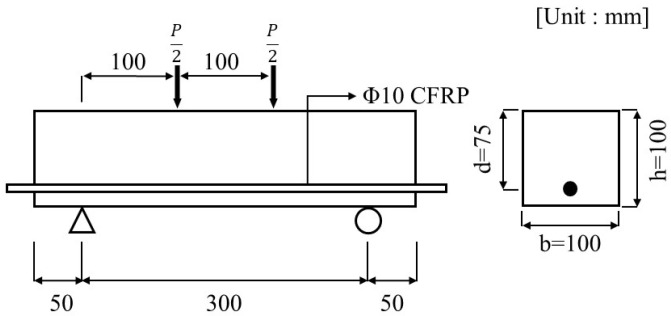
Details of carbonation specimen.

Carbonation specimens were fabricated in accordance with KS F 2584 [23], with dimensions of 400 mm in length, 100 mm in height, and 100 mm in width, as illustrated in Figure 6. The flexural specimens were reinforced with CFRP at the center, with a cover thickness of 25 mm.

### 3.2. Experiment Method

#### 3.2.1. Chloride Environment Exposure Test

**Figure 7:**
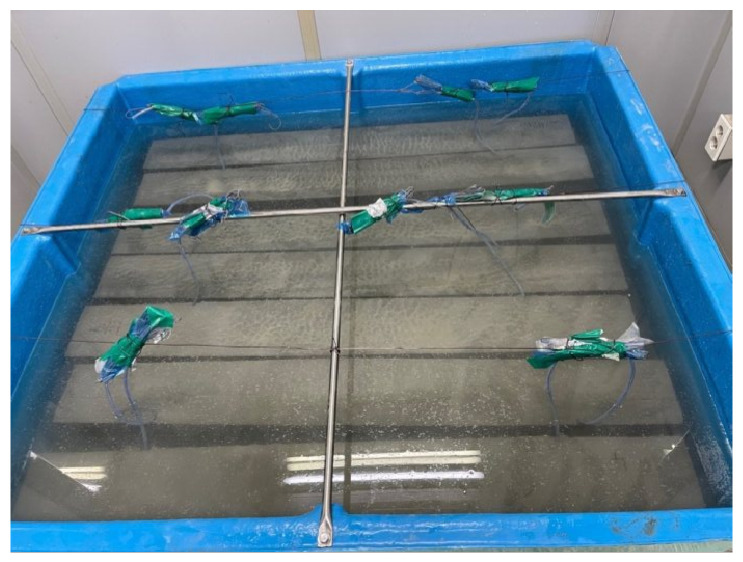
Chloride specimens in 10% NaCl solution.

Chloride exposure duration was designated as a variable to assess the durability of concrete specimens reinforced with CFRP rebar in a chloride environment. The exposure periods were 35 and 70 d, with two specimens fabricated for each duration. Similarly to a marine environment, the specimens were immersed in a container with a 10% NaCl solution, as shown in Figure 7. The temperature was maintained at room temperature (20 °C), and to prevent changes in concentration due to evaporation, the container was refilled with water throughout the immersion period.

#### 3.2.2. Accelerated Carbonation

**Figure 8:**
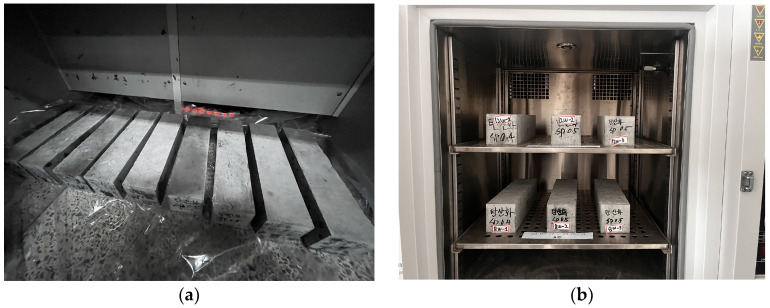
Carbonation specimens in carbonation chamber. (**a**) Carbonation specimen; (**b**) carbonation.

For the carbonation test, the specimen underwent underwater curing for four weeks, followed by maintenance in a controlled temperature and humidity chamber until it reached eight weeks of age. The specimen was then subjected to carbonation testing for 8 and 12 weeks at a temperature of 20 °C, a relative humidity of 60%, and a carbon dioxide concentration of 5%. Figure 8a depicts the carbonated specimen, while Figure 8b presents an overview of the specimen inside the carbonation chamber.

### 3.3. Experiment Results

#### 3.3.1. Durability of Specimen Exposed to Chloride Environment

**Figure 9:**
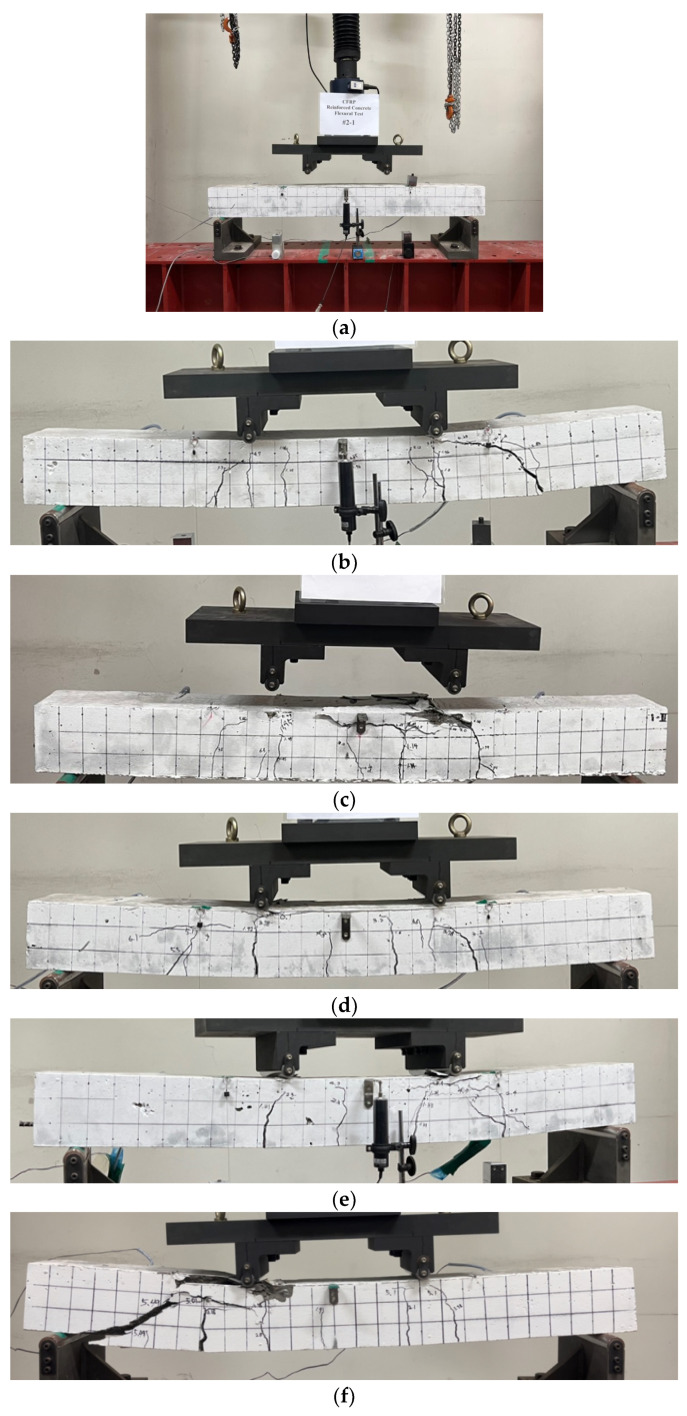

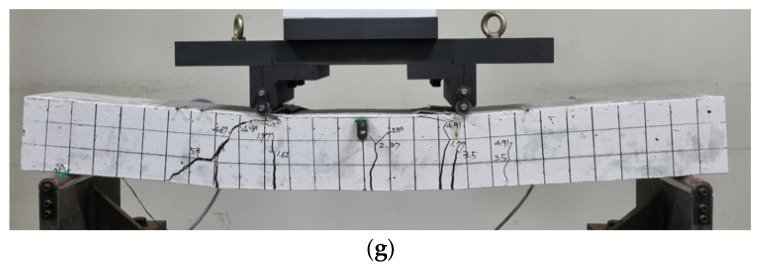
Flexural strength test results for chloride specimen. (**a**) Flexural test set-up for chloride specimen; (**b**) test result of CHP-1; (**c**) test result of CHP-2; (**d**) test result of CH35-1; (**e**) test result of CH35-2; (**f**) test result of CH70-1; (**g**) test result of CH70-2.

To assess the durability of concrete specimens reinforced with CFRP rebar in a chloride environment, a four-point loading test was conducted, as shown in Figure 9a. The loading rate was 0.5 mm/min, and two strain gauges were embedded in the D10 carbon reinforcement to measure strain in the pure flexural section. As a result, flexural failure occurred in most specimens, as shown in Figure 9b–g.

**Figure 10:**
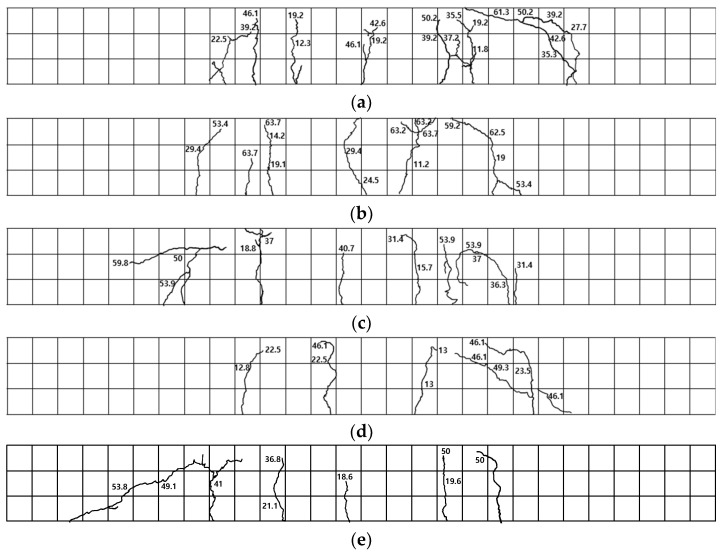

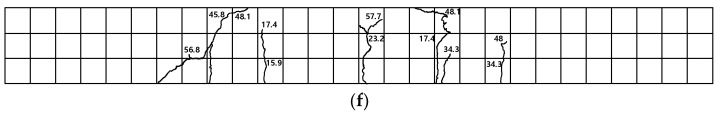
Failure modes of chloride specimens. (**a**) CHP-1; (**b**) CHP-2; (**c**) CH35-1; (**d**) CH35-2; (**e**) CH70-1; (**f**) CH70-2.

The failure modes of the flexural strength test for the chloride specimens are illustrated in Figure 10. For specimen CHP-1, as shown in Figure 10a, initial cracks occurred in the flexural section, and characteristics of flexural failure, including bending–shear cracks between the pure flexural section and the support, as well as bending cracks within the pure flexural section, were observed. For specimen CHP-2, as shown in Figure 10b, flexural cracks developed in the pure flexural section, followed by compressive failure in the upper portion of the concrete.

Specimen CH35-1, exposed to a chloride environment for 35 d, exhibited flexural cracking, as depicted in Figure 10c, with cracks propagating upwards and resulting in concrete crushing at the loading region. Specimen CH35-2 showed early cracking in the pure flexural section, as illustrated in Figure 10d, followed by a flexural–shear crack on the lower left side of the right section.

Specimen CH70-1, exposed to a chloride environment for 70 d, exhibited shear failure after flexural cracking in the pure flexural section, without reaching the upper section of the concrete, as shown in Figure 10e. Specimen CH70-2 showed initial cracking in the lower left section, followed by flexural cracks in the central region and lateral sides, which progressed upwards.

#### 3.3.2. The Durability of the Carbonated Specimen

**Figure 11:**
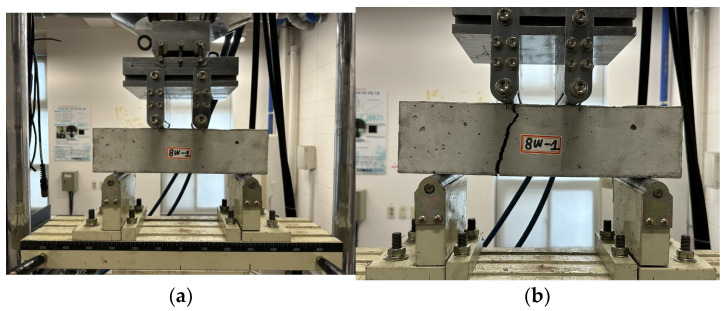
Flexural strength test set-up for carbonation specimen. (**a**) Test set-up; (**b**) result of carbonation flexural strength test.

The flexural strength test for the carbonated specimen was conducted using a four-point loading method at a rate of 5 mm/min, as shown in Figure 11a. Each specimen experienced flexural failure, as depicted in Figure 11b.

## 4. An Analysis of the Durability Test Results for Concrete Reinforced with CFRP Rebar

### 4.1. Chloride Exposure

#### 4.1.1. Load–Deflection for Chloride Specimens

**Figure 12:**
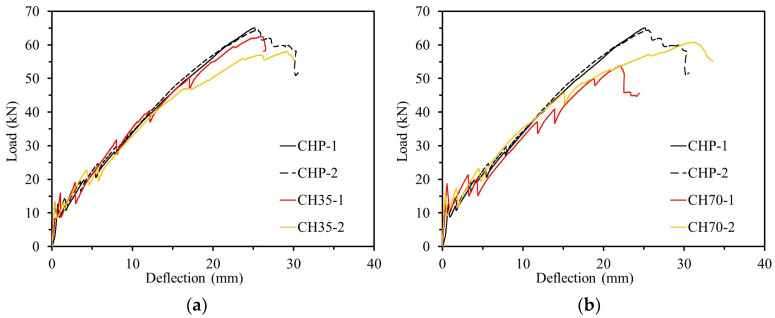
Load–deflection relationship of chlorine experiment. (**a**) Specimens exposed for 35 days; (**b**) specimens exposed for 70 days.

Table 4 presents the durability test results of specimens exposed to a chloride environment. In the flexural strength test, the ultimate load for the plain variant was measured at an average of 64.76 kN. Exposure to a chloride environment for 35 d resulted in a reduction of approximately 7.00% in ultimate load compared to the plain variant. Specimens exposed for 75 d showed an 11.46% reduction compared to the plain variant. This decrease in ultimate load was attributed to the penetration of calcium chloride into the CFRP rebar and concrete, which degraded its performance. Figure 12 illustrates the load–deflection graph for specimens exposed to a chloride environment. As shown in Figure 12, an increase in the duration of exposure correlates with a reduction in the ultimate load-bearing capacity of concrete reinforced with CFRP rebar. Additionally, exposure to a chloride environment resulted in an increase in deflection by 4.38% to 6.54% compared to the plain variant. This increase in deflection is attributed to the fact that, despite the decrease in the durability of concrete structures reinforced with CFRP when exposed to a chloride environment over time, the CFRP reinforcement’s excellent corrosion resistance and complete adhesion to the concrete help resist loads. This increased deflection helps prevent brittle failure.

#### 4.1.2. Load–Strain Relation for Chloride Specimens

**Figure 13:**
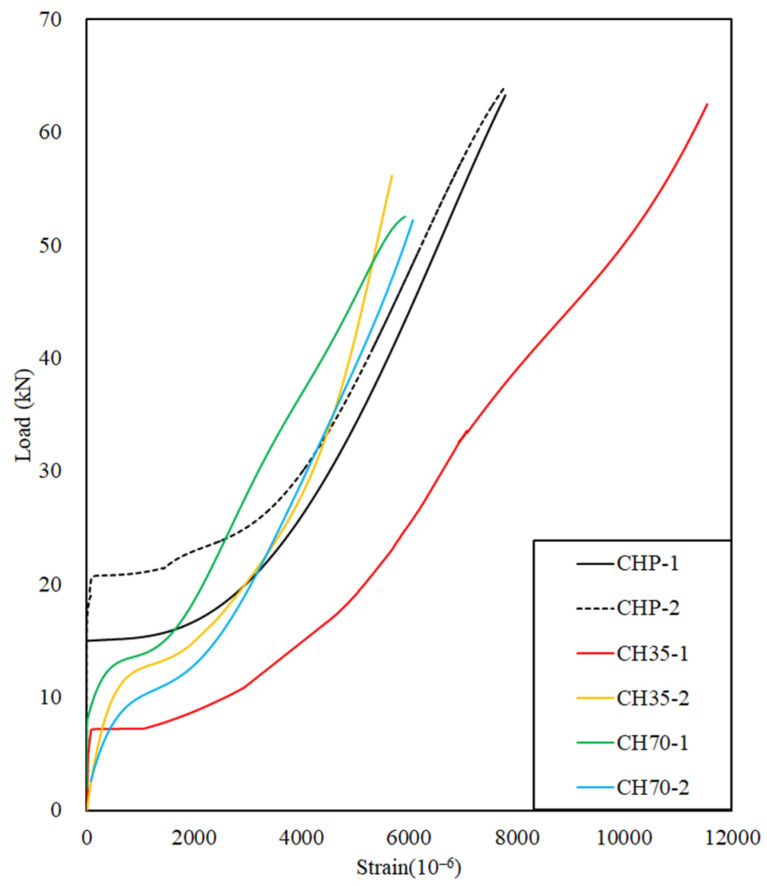
Load–strain relationship of chloride during flexible strength test.

Figure 13 shows the load–strain curve of the CFRP rebar. Specimen CHP-1 began initial deformation at 15 kN, while CHP-2 deformation started at 21 kN, with strain increasing linearly until fracture occurred at approximately 64 kN.

Specimen CH35-1 began initial deformation at 6.5 kN, exhibiting a lower slope compared to the CHP specimens, resulting in greater strain, and it ultimately fractured at 62 kN.

Specimen CH35-2 showed early deformation, with deflection occurring at 12 kN and failure at 55 kN.

In a chloride environment, after 35 d of exposure, the ultimate load value decreased by approximately 9.58% compared to the CHP specimens. CH70 specimens exposed to a chloride environment for 70 d exhibited a reduction of approximately 19.26% in ultimate load values compared to the CHP specimens.

CH35 exhibited a maximum strain increase of 10.62% compared to CHP specimens owing to the CFRP rebar, which, despite being exposed to a chloride environment, appears to prevent brittle failure in the concrete by increasing the strain in the CFRP rebar due to its complete adhesion to the concrete, thereby enhancing load resistance.

CH70 showed a decrease in both extreme load and maximum strain compared to the CHP specimens. When the extreme load value of the CH70 specimen reached 52.29 kN, the strains of the CHP-1 and CHP-2 specimens were 5380.48 mm/mm and 6777.14 mm/mm, respectively. The strains of CHP-1 and CHP-2 compared to CH70 were approximately −9.77% and +13.65%, respectively, owing to the deterioration in concrete durability caused by exposure to the chloride environment, resulting in reduced strength. Despite this, CFRP rebar continues to perform effectively in its tensile function even in chloride environments, demonstrating no significant degradation in durability.

#### 4.1.3. Ductility Index

Ductility is defined as the change in the slope of the load–displacement curve and the amount of energy absorbed. The ductility measure is usually defined as a ratio using displacement at the ultimate load and displacement at the yield point, which is called the ductility index (μ).

FRP does not undergo inelastic deformation due to brittle fracture without a yield point, but it only releases elastic strain energy. This results in no change in the slope of the load–displacement curve and immediate failure.

**Figure 14:**
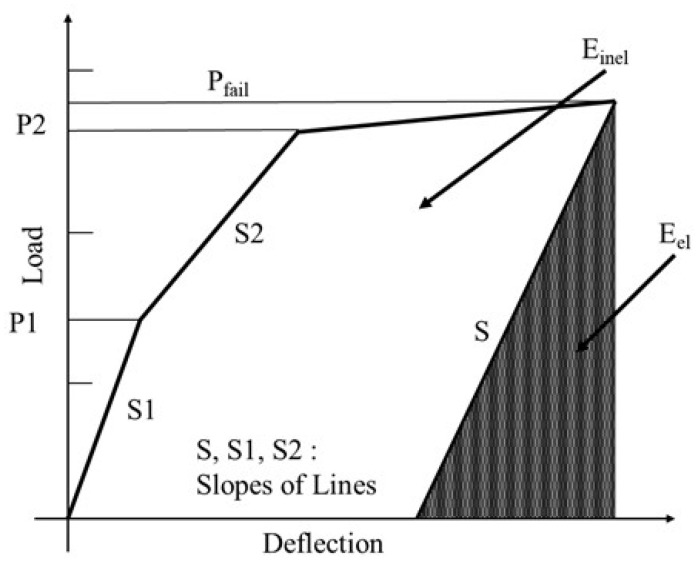
Definition of ductility index; Naaman and Jeong [24].

Naaman and Jeong [24] proposed the ductility index equation with an energy-based model to evaluate the ductility of FRP-reinforced concrete, and their method was employed in this study. The ductility index is not defined simply as the ratio of deflection, but as the ratio of elastic energy (E_el_) to inelastic energy (E_inel_). Here, in Figure 14, the elastic energy can be estimated from unloading experiments, or in the absence of data, it is calculated as the area of the triangle formed by the weighted average slope of the first two straight sections of the load–deflection curve.

The ductility index can be computed using Equation (3) and presented in Table 5.

(3) $$\mu =\frac{1}{2}\left(\frac{E_{\mathrm{el}}+E_{\mathrm{inel}}}{E_{\mathrm{el}}}+1\right)$$

The average ductility indexes tend to decrease in the order of CHP, CH35, and CH70. This means that the amount of elastic and inelastic energy absorption varies depending on the duration of exposure to the salt environment, which means that the ductility of the structure decreases and the tendency of brittle behavior increases.

#### 4.1.4. SEM Analysis for Chloride Conditions

**Figure 15:**
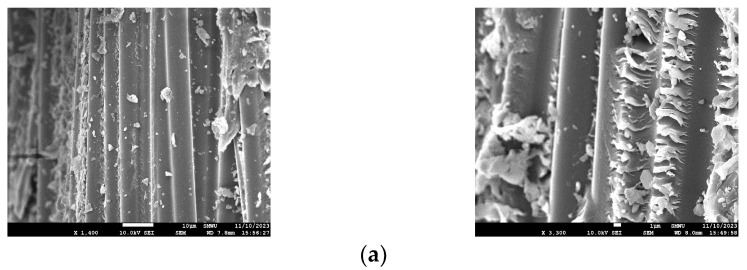

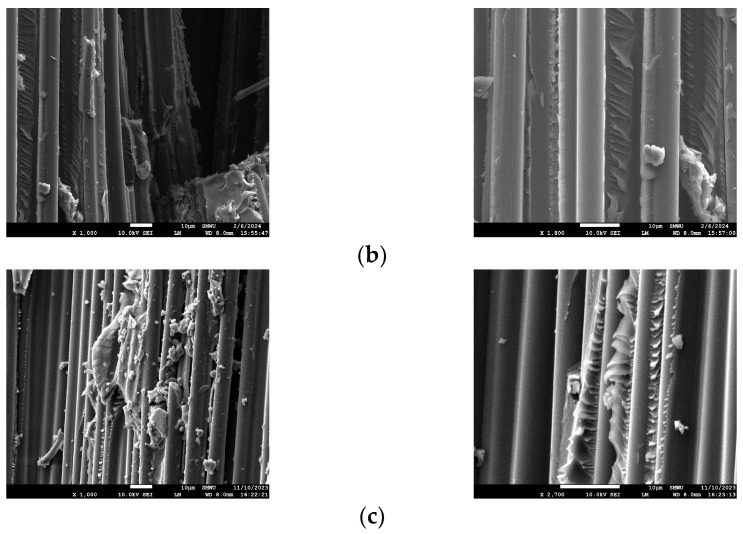
SEM images of chloride specimens. (**a**) CHP SEM; (**b**) CH35 SEM; (**c**) CH70 SEM.

The microstructure of D10 CFRP reinforcement exposed to chloride was analyzed using scanning electron microscopy (SEM). Figure 15a–c depict the specimens CHP, CH35, and CH70, respectively. All specimens display a mixture of carbon fiber, resin, and concrete. An SEM analysis revealed no damage to the fibers in any of the specimens, confirming that chlorine exposure did not impact the durability of the CFRP rebar.

### 4.2. Carbonation

#### 4.2.1. Load–Deflection for Carbonation Specimens

**Figure 16:**
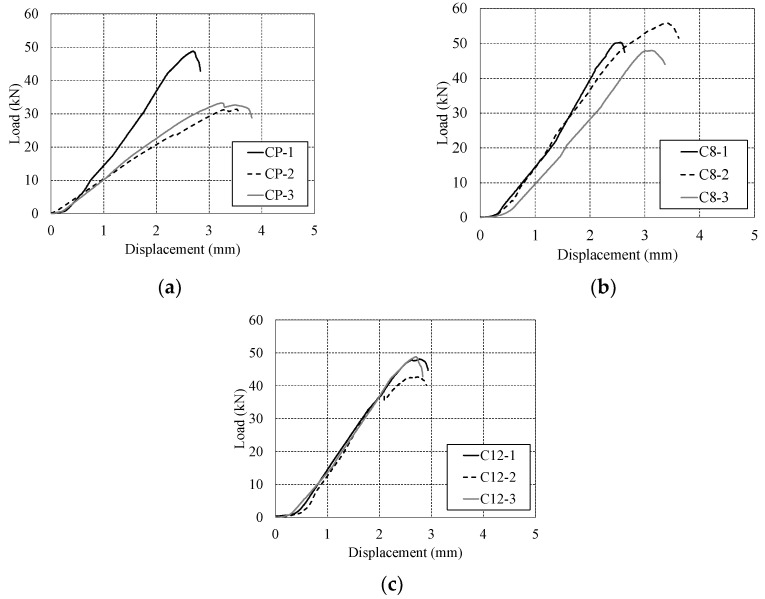
Load–deflection relationship during carbonation flexural strength test. (**a**) Plain variant; (**b**) carbonation for 8 weeks; (**c**) carbonation for 12 weeks.

The flexural strength test results of the specimens subjected to accelerated carbonation for 8 and 12 weeks are shown in Figure 16 and Table 6. Figure 16a depicts the CP specimen, which was not subjected to carbonation, with a maximum load of 37.80 kN. The load–deflection relationship within the specimen after 8 weeks of carbonation is shown in Figure 16b, with an average maximum load of 51.34 kN. Figure 16c presents the load–deflection relationship in the specimen after 12 weeks of carbonation, with an average maximum load of 47.19 kN. The loads of the specimens that underwent 8-week and 12-week carbonation periods increased by approximately 35.82% and 24.84%, respectively, compared to the specimens that did not undergo carbonation. In general, since the CP specimens were not subjected to carbonation, higher load values were expected compared to the C8 and C12 specimens. However, it is believed that the performance of the CP specimens was not fully realized due to insufficient curing of the concrete. Nonetheless, as carbonation progressed, a load reduction of 9.01% was observed, indicating a decrease in the strength of the concrete.

#### 4.2.2. Carbonation Depth

**Figure 17:**
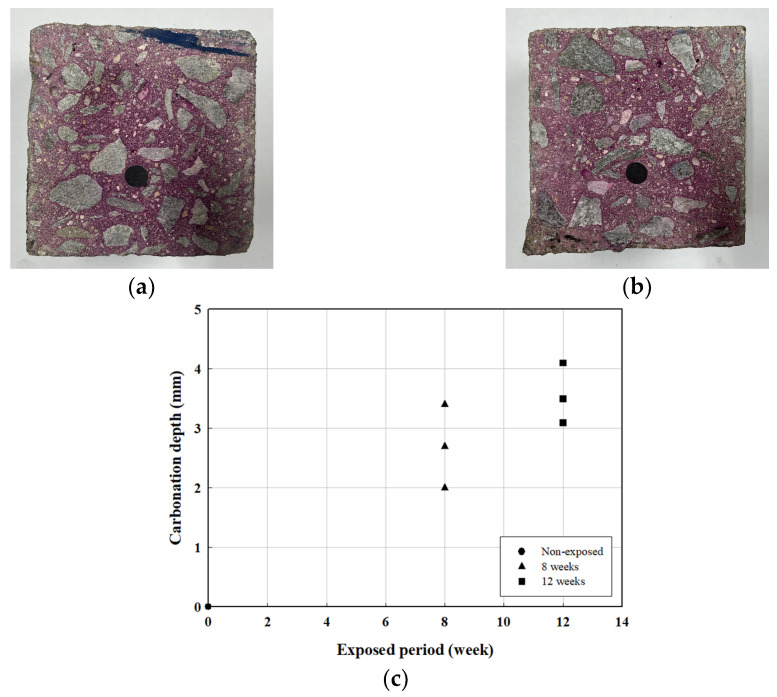
Carbonation depth measurements. (**a**) C8; (**b**) C12; (**c**) carbonation depth according to exposure period.

The carbonation depth of the specimen was measured according to the carbonation process. Measurements were conducted in accordance with KS F 2596 [25]. Figure 17a,b show the application of phenolphthalein solution on the fractured surfaces of the C8 and C12 specimens for carbonation depth measurement, while Figure 17c illustrates the carbonation depth over time. Table 7 presents the results of the carbonation depth and rate measurements. For carbonation prediction, Equation (4) was used [26,27,28], and the carbonation rate was determined using

(4) $$C=A\sqrt{t}$$

where A is the speed factor, t is the carbonation period, and C is the carbonation depth.

Both the carbonation depth and rate of the concrete increased with the progression of carbonation.

#### 4.2.3. SEM Analysis for Carbonation

**Figure 18:**
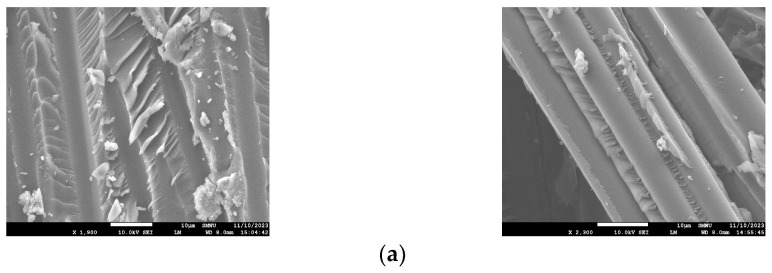

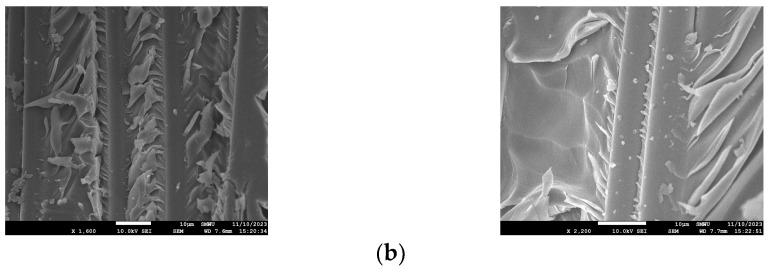
SEM images of specimens undergoing carbonation. (**a**) C8 SEM; (**b**) C12 SEM.

An SEM analysis was performed to observe the microstructural changes in the CFRP rebar of the carbonated specimens, and the results are shown in Figure 18.

The analysis confirmed that the concrete adhered to the CFRP fibers, as shown in Figure 18a,b, and that no adverse effect was observed on the CFRP fibers as carbonation progressed.

## 5. Conclusions

This study evaluated the durability of concrete reinforced with D10 CFRP rebar through chloride exposure experiments and carbonation tests.

Chloride-exposed CFRP rebar-reinforced concrete beams underwent flexural strength testing, with most specimens experiencing flexural failure. Regarding the load–deflection relationship in the chloride specimens, the initial crack load of CH35 and CH70 increased, and regarding the load–strain relationship, the strain values for CH35 and CH70 were higher compared to the CHP specimen when resisting the same load. These results indicate that exposure to chloride does not compromise the durability of CFRP reinforcement. Additionally, an SEM analysis of the chloride specimens showed no deterioration of the D10 carbon reinforcement after 35 and 70 d of chloride exposure.

Regarding the load–deflection relationship of the carbonation specimens, strength decreased while deflection increased as carbonation progressed. Supposedly, the progression of carbonation could cause a reduction in the concrete’s bond strength. However, the increased deflection allows the CFRP reinforcement to resist the load and prevent brittle failure in the concrete. The average ductility indexes tend to decrease. This means that the amount of elastic and inelastic energy absorption varies depending on the duration of exposure to the salt environment, which means that the ductility of the structure decreases and the tendency of brittle behavior increases. An SEM analysis confirmed that accelerated carbonation for 8 and 12 weeks did not cause degradation in the D10 CFRP reinforcement, and there was no remarkable reduction in its durability. In the future, the long-term durability of CFRP rebar will be evaluated in extreme environments by extending both the chloride exposure and carbonation periods.

## Figures and Tables

**Table 1:** Tensile strength test results of CFRP rebar.

Description	Ultimate Load (kN)	Tensile Stress (MPa)	Strain (mm/mm)	Modulus of Elasticity (GPa)
CFRP-1	193.2	2461.3	0.0179	135.7
CFRP-2	183.4	2336.2	0.0169	141.4
CFRP-3	175.8	2239.9	0.0174	135.6
CFRP-4	185.8	2367.4	0.0197	121.8
CFRP-5	176.5	2249.2	0.0157	137.2
Average	182.9 ± 6.4	2330.8 ± 81.6	0.0175 ± 0.001	134.3 ± 6.6

**Table 2:** Mix proportions of test specimens.

Slump (mm)	Air Contents (%)	W/C ^1^ (%)	S/a ^2^ (%)	Unit Weight				
				W (kg) ^3^	C ^4^	SF ^5^	S ^6^	G ^7^
43	2	33	45	165	475	25	755	905

^1^ Water-to-cement ratio. ^2^ Fine aggregate ratio. ^3^ Water. ^4^ Cement. ^5^ Silica Fume. ^6^ Sand. ^7^ Coarse aggregates.

**Table 3:** Results of compressive strength test for concrete.

No.	Compressive Strength (MPa)	Modulus of Elasticity (GPa)
C-1	55.70	33.50
C-2	54.90	33.30
C-3	51.40	32.60
C-4	59.60	34.20
C-5	59.60	34.20
Average	56.24 ± 3.1	33.56 ± 0.6

**Table 4:** Chloride experiment results.

Specimen Identification	P_cr_ (kN)	∆_cr_ (mm)	P_ultimate_ (kN)	∆_u_ (mm)
CHP-1	12.50	0.76	65.08	25.11
CHP-2	12.89	0.39	64.44	25.59
Average	12.70	0.57	64.76	25.35
CH35-1	15.40	1.29	62.45	24.86
CH35-2	13.45	0.42	58.01	29.15
Average	14.4	0.42	60.23	27.01
CH70-1	18.69	0.22	53.83	21.74
CH70-2	15.83	0.37	60.80	31.18
Average	17.26	0.29	57.34	26.46

**Table 5:** The ductility index for the specimens exposed to chloride conditions.

Specimen Designation	E_inel_ (kN·mm)	E_el_ (kN·mm)	E_tot_ (kN·mm)	*μ*
CHP-1	535	423	958	1.63
CHP-2	346	664	1010	1.26
CH35-1	484	528	1011	1.46
CH35-2	490	665	1155	1.37
CH70-1	62	672	734	1.05
CH70-2	715	695	1410	1.51

**Table 6:** Results of carbonation test.

Specimens	Max Load (kN)	Deflection at Max Load (mm)
CP-1	48.82	2.70
CP-2	31.38	3.53
CP-3	33.21	3.23
Average	37.80 ± 7.83	3.15
C8-1	50.29	2.55
C8-2	55.80	3.41
C8-3	47.92	3.12
Average	51.34 ± 3.30	3.02
C12-1	48.12	4.91
C12-2	44.62	3.51
C12-3	48.82	2.70
Average	47.19 ± 1.84	3.70

**Table 7:** Carbonation depth and speed factor.

Specimens	Curing (Weeks)	Carbonation Depth (C, mm)	$A\left(C/\sqrt{\mathrm{weeks}}\right)$
C8-1	8	2.0	0.7
C8-2		2.7	1.0
C8-3		3.4	1.2
Average		2.70	0.97
C12-1	12	3.1	0.9
C12-2		4.1	1.2
C12-3		3.5	1.0
Average		3.57	1.03

## Data Availability

The original contributions presented in the study are included in the article, further inquiries can be directed to the corresponding author.
